# Increased leptin signaling drives the response of hypothalamic LepRb neurons to diet-induced obesity

**DOI:** 10.1016/j.molmet.2026.102378

**Published:** 2026-05-02

**Authors:** James Dell'Orco, Warren Pan, Margaret B. Allison, Abigail J. Tomlinson, Jordan Wean, Paul V. Sabatini, Christopher J. Rhodes, David P. Olson, Martin G. Myers Jr., Paulette B. Goforth

**Affiliations:** 1 Department of Internal Medicine, University of Michigan, Ann Arbor, MI, USA; 2 Department of Pharmacology, University of Michigan, Ann Arbor, MI, USA; 3 Graduate Program in Cellular and Molecular Biology, University of Michigan, Ann Arbor, MI, USA; 4 Department of Molecular and Integrative Physiology, University of Michigan, Ann Arbor, MI, USA; 5 Department of Surgery, University of Michigan, Ann Arbor, MI, USA; 6 Department of Medicine, University of Chicago, Chicago, IL, USA; 7 Division of Endocrinology, Department of Pediatrics, University of Michigan, Ann Arbor, MI, USA

**Keywords:** Leptin, Hypothalamus, Obesity, Gene expression, Neuron excitability

## Abstract

The failure of hyperleptinemia to decrease adiposity in common forms of obesity has led to the notion that impaired leptin receptor (LepRb) signaling (“leptin resistance”) might cause obesity. Because LepRb transcriptional signaling plays a central role in leptin action, we defined the control of gene expression in hypothalamic LepRb neurons in diet-induced obese (DIO) mice and in response to changes in circulating leptin. We found that LepRb neurons from DIO mice exhibited transcriptional changes similar to those induced by exogenous leptin. We also examined electrical activity in LepRb neurons from DIO mice, focusing on LepRb neurons in the ventromedial hypothalamic nucleus (VMN). This analysis revealed larger membrane depolarizations in response to current injection for VMN LepRb neurons from DIO mice. This effect was recapitulated by hyperleptinemia *in vivo* or exposure to elevated leptin *ex vivo*. Hence, hypothalamic LepRb neurons exhibit increased cellular leptin responses due to hyperleptinemia in DIO animals. These findings contradict the notion that impaired cellular leptin action underlies the development of DIO but rather suggest that increased leptin action drives DIO-associated changes in hypothalamic LepRb neuron function.

## Highlights

• Appropriate transcriptional responses to hyperleptinemia in LepRb neurons in DIO mice.
• VMN LepRb neurons from DIO mice display leptin-mediated hyperexcitability.
• No evidence for diminished cellular leptin action in DIO mice.

## 1 Introduction

White adipose tissue produces the hormone, leptin, in proportion to triglyceride stores to signal the repletion of body energy stores [1,2]. Leptin acts in the brain to suppress feeding, promote energy expenditure, and support endocrine function. Although alternative splicing gives rise to other leptin receptor (LepR) isoforms, the long (LepRb) form of the receptor is necessary and sufficient for leptin action. Consistent with the role for LepRb in energy balance, most LepRb-containing cells represent specialized subsets of neurons in brain areas that contribute to the control of food intake and energy expenditure.

Leptin-mediated activation of anorexigenic proopiomelanocortin (POMC)-containing neurons of the hypothalamic arcuate nucleus (ARC) contributes to the control of food intake and energy balance [[1], [2], [3], [4], [5]], as does the direct and indirect (via GABAergic *Lepr* neurons marked by the expression of *Glp1r*, *Trh*, or *Bnc2*) inhibition of orexigenic neuropeptide Y/agouti-related peptide (NPY/AgRP)-containing neurons [[6], [7], [8], [9]]. Direct leptin action on other sets of hypothalamic LepRb neurons, including in the ventromedial hypothalamic nucleus (VMN), promote energy expenditure [10,11]. Additional populations of hypothalamic LepRb neurons also likely contribute to the control of energy balance by leptin [4].

Leptin binding activates the LepRb-associated Janus kinase 2 (JAK2) tyrosine kinase, which phosphorylates intracellular tyrosine residues on the receptor to promote the recruitment of downstream signaling proteins [12,13]. Phosphorylated LepRb Tyr_1138_ recruits the transcription factor, STAT3, thereby permitting its tyrosine phosphorylation (pSTAT3) and transcriptional activation. Mice lacking LepRb Tyr_1138_ or ablated for STAT3 in LepRb neurons display hyperphagic obesity, revealing a crucial role for STAT3-mediated transcriptional control in leptin action [[12], [13], [14], [15]]. Leptin also alters the electrical activity of many LepRb neurons-increasing the firing of many POMC cells and VMN LepRb neurons, in addition to decreasing the activity of NPY/AgRP cells [10,[16], [17], [18]].

While endogenous leptin signaling is required to prevent obesity and exogenous leptin decreases body weight in leptin-deficient or lean animals, elevated circulating leptin concentrations in common forms of obesity (such as diet-induced obesity (DIO)) fail to promote weight loss [19,20]. Furthermore, administering exogenous leptin to hyperleptinemic DIO animals minimally augments pSTAT3 and fails to promote significant weight loss, suggesting that impaired LepRb signaling might underlie obesity [21]. Many thus refer to obesity as a state of “leptin resistance”.

Several processes have been suggested to mediate this postulated impairment of LepRb signaling during DIO, including impaired leptin transport into the CNS, increased inflammatory signaling, endoplasmic reticulum (ER) stress, and the expression of cellular inhibitors of LepRb signaling (including *Socs3*, *Ptpn1*, and *Ptpn2*) in LepRb neurons [[22], [23], [24], [25], [26], [27], [28], [29], [30]]. Conversely, recent studies have suggested that increased leptin action in obesity might contribute to the complications of obesity [18,[31], [32], [33], [34], [35]].

We thus sought to understand the physiology of LepRb neurons in DIO mice. Because LepRb neurons comingle with many other cell types in the brain, we analyzed mRNA specifically from hypothalamic LepRb neurons in leptin-treated and DIO mice. We also used electrophysiology to define DIO-and leptin-induced changes in electrical activity in genetically identified VMN LepRb neurons. In contrast to what might be predicted by the theory that “leptin resistance” causes obesity, we found that the examined LepRb neurons do not display decreased transcriptional and electrical responses to leptin, but rather exhibit changes that reflect increased cellular LepRb signaling, suggesting that DIO-induced hyperleptinemia promotes a state of increased cellular leptin action in at least some hypothalamic LepRb neurons.

## 2 Methods

### 2.1 Animals

All procedures performed on animals were approved by the University of Michigan Institutional Committee on the Care and Use of Animals and in accordance with AALAC and NIH guidelines. All mice were provided with water *ad libitum* and housed in temperature-controlled rooms on a 12-hour light–dark cycle. All mice were provided *ad libitum* access to standard chow diet (Purina Lab Diet 5001) or HFD (Research Diets D12492, 60% from fat), unless otherwise noted.

All mice were bred in our colony in the Unit for Laboratory Animal Management at the University of Michigan. *Lepr*^*cre/cre*^*;Rosa*^*eGFP−10a/eGFP-L10a*^ (LepRb^eGFP−L10a^) and *Lepr*^*cre/cre*^*;Slc16a2*^*Flp/Flp*^ mice were as previously described [11,36]. Mice were weaned at 21 days and group housed with same-sex littermates fed the appropriate diet.

Mice were weighed prior to euthanasia; any HFD-fed mice not meeting criteria for obesity (weight >28 g for females; weight >30 g for males) were removed from study. Some mice were treated with metreleptin (5 mg/kg, i. p., 10 h; AstraZeneca) or with vehicle (0.9% saline, 10 h, Hospira, i. p.).

Some 10-12 week-old chow-fed LepRb^eGFP−L10a^ mice were implanted subcutaneously with osmotic minipumps (Alzet Model 1002; Alza, Palo Alto, CA) filled with either leptin (metreleptin, 2.2 mg/kg per day; AstraZeneca) or vehicle (0.9% sodium chloride, Hospira) and were single housed for 10–11 days before euthanasia and tissue dissection.

### 2.2 Translating ribosome affinity purification with deep sequencing (TRAP-seq)

At the midpoint of the light cycle, adult mice were anesthetized with isofluorane, after which their brains were removed and placed onto a mouse coronal brain matrix (1 mm sections). For whole hypothalamic dissections, a 3 × 3x3mm block was dissected from the ventral diencephalon immediately caudal to the optic chiasm and immediately homogenized and processed for TRAP-seq analysis as previously described [36]. Hypothalamic from 5 to 8 mice were pooled at the time of collection to produce sufficient mRNA for a single RNA-seq replicate.

Messenger RNA isolated from eGFP-tagged ribosomes and from the eGFP-depleted supernatant was assessed for quality using TapeStation (Agilent, Santa Clara, CA) and samples with RNA Integrity Numbers (RINs) of 8 or greater were prepared using the Illumina TruSeq mRNA Sample Prep v2 kit (Catalog # RS-122-2001 and #RS-122-2002) (Illumina, San Diego, CA), where 0.1–3 μg of RNA was converted to mRNA using a polyA purification. The mRNA was chemically fragmented and copied into first strand cDNA using reverse transcriptase and random primers. The 3′ ends of the cDNA were adenylated and the 6-nucleotide-barcoded adapters ligated. These products were then purified and enriched by PCR to create cDNA libraries, which were checked for quality and quantity by TapeStation (Agilent) and qPCR using Kapa's library quantification kit for Illumina Sequencing platforms (catalog #KK4835) (Kapa Biosystems, Wilmington MA). They were clustered on cBot (Illumina) and sequenced 4 samples per lane on a 50-cycle single end run on a (Illumina) using version 2 reagents according to manufacturer's protocols.

### 2.3 RNA-seq analysis

50 base pair single end reads underwent QC analysis (FastQC) prior to quality filtering using fastq_quality_filter 0.0.14 with q ≥ 20 and alignment to mouse genome build GRCm38 (mm10) using STAR 2.5.3a_modified. Differential expression was determined using DESeq2 1.22.2 in R 3.5.2. Whole hypothalamic DIO samples were compared against previously-reported data [37] for chow-fed animals to determine changes in gene expression; PEG-SMLA-treated samples were compared against 20-hour PBS-treated controls to determine changes in gene expression. Changes in gene expression for leptin-treated (10 h) and *ob/ob* animals were previously reported [37,38].

### 2.4 Preparation of brain slices for electrophysiological recordings

LepRb^eGFP−L10a^ mice were anesthetized by isoflurane inhalation, decapitated, and the brains were chilled in ice-cold cutting solution containing (in mM): 130 NaCl, 3 KCl, 26 NaHCO_3_, 1.25 NaH_2_PO_4_, 2.5 CaCl_2_, 1 MgCl_2_, 1 kynurenic acid, 0.4 ascorbic acid, 5 sucrose, and 2 glucose, bubbled with 5% CO_2_/95% O_2_. 250 μm coronal slices were cut using a vibratome and incubated for 45 min at 33 °C in artificial CSF (ACSF) containing (in mM): 130 NaCl, 3 KCl, 26 NaHCO_3_, 1.25 NaH_2_PO_4_, 2.5 CaCl_2_, 1 MgCl_2_ and 2 glucose, bubbled with 5% CO_2_+ 95% O_2_ (carbogen) then shifted to room temperature until recording. For *ex vivo* leptin pre-incubation, slices were incubated in ACSF containing 100 nM leptin, which remained throughout electrophysiological recordings. Following incubation/recovery, slices were placed in a recording chamber and continuously perfused with carbogen-bubbled ACSF at 33 °C with a flow rate of 2–3 ml/min.

### 2.5 Whole-cell recordings

GFP-containing neurons in the VMN were identified for patch-clamp recordings in coronal slices. Borosilicate glass patch pipettes (with resistances of 3–4 MΩ) were filled with internal recording solution containing (in mM): 119 K Gluconate, 2 Na Gluconate, 6 NaCl, 2 MgCl_2_, 0.9 EGTA, 10 HEPES, 14 Tris-Phosphocreatine, 4 MgATP, 0.3 tris-GTP, pH 7.3. Whole-cell recordings were performed 1–5 h after slice preparation using an Axopatch 700 B amplifier, Digidata 1550 A interface and pClamp-10.5 software (MDS analytical technologies, Sunnyvale, CA). Voltage measurements were acquired in the fast current clamp mode of the amplifier necessary to reduce distortion of action potentials and filtered at 2 kHz. [39]. Series resistance was compensated for up to 60% when possible. Cells were rejected if the series resistance changed by greater than 20% during the recording or if it exceeded 25 MΩ. Total cell capacitance was calculated from measurement of the area under capacitive current transients evoked from a 10 mV depolarizing step from −80 mV, following correction for input resistance. Data were digitized at 100 kHz. Voltage traces were corrected for a calculated 10 mV liquid junction potential. Input-output curves were generated by injecting a sufficient amount of negative current to hold the cells at −60 mV and subsequently delivering successive 1 s duration square waveform current pulses ranging from −60 to +120 pA of injected current at 10 pA successive intervals.

### 2.6 DIO DREADD study

*Lepr*^*Cre*^*;Slc17a6*^*Flp*^ mice were anesthetized with isoflurane (2%) and mounted in a stereotaxic frame (Kopf). Using standard surgical techniques, 150 nL of virus (AAV-hSYN1-fDIO-tTA and AAV-TRE-DIO-hM3Dq-mCherry in equal proportions) was injected bilaterally via a glass micropipette attached to a microinjector (picospritzer II) targeting the VMN (AP -1.3 mm; ML ±0.25 mm, DV -5.55 mm, relative to bregma). Following surgery, mice were placed on HFD for 8 or more weeks. Animals were singly housed for one week prior to study. To determine the effect of activating VMN LepRb neurons on food intake and body weight in DIO mice were injected intraperitoneally with CNO (1 mg/kg) or vehicle (0.9% Saline) twice daily. Injections were delivered at Zeitgeber time (ZT) 3 and ZT11. Body weight and food mass recorded daily.

Upon the completion of DREADD studies, mice were perfused with 10% formalin. Brains were then removed and post-fixed in 10% formalin for 24 h before being moved to 30% sucrose for 24 h. Brains were then sectioned as 30 μm thick free-floating sections. Immunofluorescent staining was performed using standard procedures using anti-DSRed (1:1000, #632392, Clontech) antibodies. Images were collected on an Olympus BX51 microscope. Animals demonstrating viral leak into nearby regions or demonstrating negligible transduction of VMN LepRb neurons were excluded from analysis.

### 2.7 Statistical analysis

Statistical significance for electrophysiology was assessed by either an unpaired Student's *t* test, Fisher's exact test, or Two-way ANOVA, as noted in the figure legends. Data were considered significant for p < 0.05. Data are expressed as mean ± SEM unless otherwise specified. Data was analyzed using SigmaPlot (Systat Software Inc), GraphPad Prism (GraphPad Software Inc.) and Excel (Microsoft Corp). Calculations of mean baseline spontaneous firing frequency excluded both hyperpolarized and depolarized cells that did not exhibit spontaneous action potentials.

## 3 Results

### 3.1 Leptin drives the transcriptional response to DIO in hypothalamic LepRb neurons

**Figure 1:**
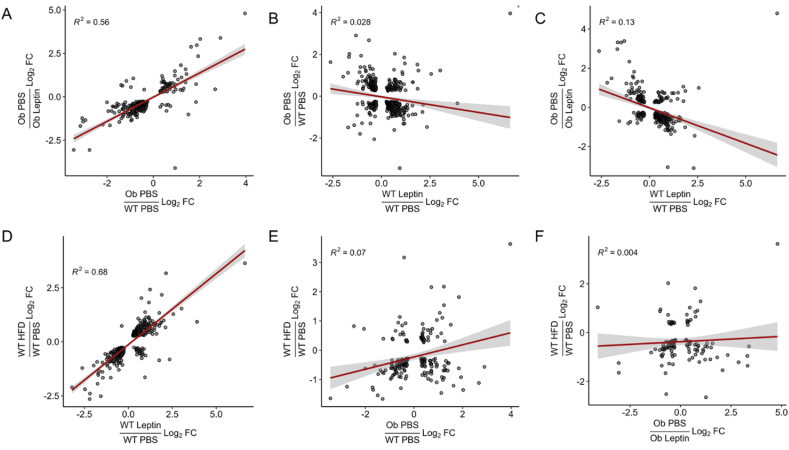
TRAP-seq analysis to determine the transcriptional response of hypothalamic LepRb neurons to DIO. Plotted are the differences in gene expression between (y-axis) (A,C) *ob/ob* mice treated with PBS vs leptin, (B) PBS-treated *ob/ob* and WT mice, or (D–F) PBS treated HFD or Chow-fed WT mice compared to (x-axis) differences in gene expression between (A, E) PBS-treated *ob/ob* and WT mice, (B–D) leptin and PBS-treated WT mice, (E) PBS-treated *ob/ob* and WT mice, (F) *ob/ob* mice treated with PBS vs leptin. For all panels, log_2_Fold Change (FC) is shown for each gene that is significantly different for both comparisons.

To understand the transcriptional response of hypothalamic LepRb cells to high-fat feeding and obesity, we exposed *Lepr*^*Cre*^*;Rosa26*^*eGFP-L10a*^ (LepRb^eGFP−L10a^) mice to high-fat diet (HFD) from the time of weaning. HFD-fed mice not meeting criteria for obesity (weight >28 g for females; weight >30 g for males) were removed. Once DIO was established at 11–16 weeks of age, we harvested LepRb cell-specific mRNAs by anti-eGFP translating ribosome affinity purification (TRAP). As previously [[36], [37], [38]], we combined TRAP samples from multiple male and female mice to obtain sufficient material for RNA-seq (TRAP-seq) (Figure 1) (Supplemental Table 1).

We compared gene expression in mRNA recovered from LepRb neurons from DIO mice with that from chow-fed wild-type or leptin-deficient (*Lep*^*ob/ob*^; *ob/ob*) animals treated with vehicle or leptin (5 mg/kg, 10 h) [37] (Supplemental Tables 1–4) (Figure 1) (note that all of these studies were performed contemporaneously). Others previously showed that exogenous leptin treatment of this sort results in rapid increases in circulating leptin concentrations to over 500 ng/ml (compared to approximately 10 ng/ml for DIO animals) [40]. The overlap of significantly regulated genes between groups is shown in Supplemental Fig. 1. As expected, the changes in gene expression between vehicle-treated *ob/ob* and chow-fed controls were similar to differences in gene expression between vehicle- and leptin-treated *ob/ob* animals (Figure 1A), consistent with the notion that leptin treatment normalizes gene expression in the LepRb neurons from *ob/ob* mice. Interestingly, however, changes in gene expression induced by leptin treatment in chow-fed normal animals correlated poorly with the leptin-induced changes in gene expression in *ob/ob* mice or with changes in gene expression between vehicle-treated *ob/ob* and chow-fed wild-type animals (Figure 1B and C). This finding suggests that increasing leptin concentrations in normal lean animals promotes a gene expression program different than that induced by the normalization of leptin in leptin-deficient mice.

To understand DIO-induced alterations in LepRb neuron gene expression, we initially compared changes in gene expression between chow-fed and DIO animals with changes in gene expression between vehicle- and leptin-treated chow-fed mice (Figure 1D). This analysis revealed a strong correlation in the gene expression profiles of LepRb neurons from leptin-treated lean animals and the gene expression profiles of LepRb neurons in DIO animals, suggesting that the aggregate LepRb neuron gene expression response to DIO reflects the expected response to hyperleptinemia. These data suggest appropriately increased LepRb transcriptional signaling in DIO animals, rather than decreased LepRb signaling.

We also compared the DIO-induced differences in LepRb neuron gene expression with differences between vehicle-treated wild type and *ob/ob* mice or between vehicle- and leptin-treated *ob/ob* mice (Figure 1E and F). DIO-associated gene expression changes poorly correlated with those provoked by the normalization of leptin concentrations in *ob/ob* mice, much as the hyperleptinemia-provoked changes in gene expression in leptin-treated lean mice poorly correlated with the changes caused by leptin repletion in *ob/ob* mice (Figure 1B and C). Hence, the LepRb neuron gene expression in hyperleptinemic DIO animals closely resembles gene expression in hyperleptinemic lean animals. The gene expression changes promoted by exogenous or endogenous hyperleptinemia are different from those promoted by the normalization of leptin in leptin-deficient animals, however.

**Figure 2:**
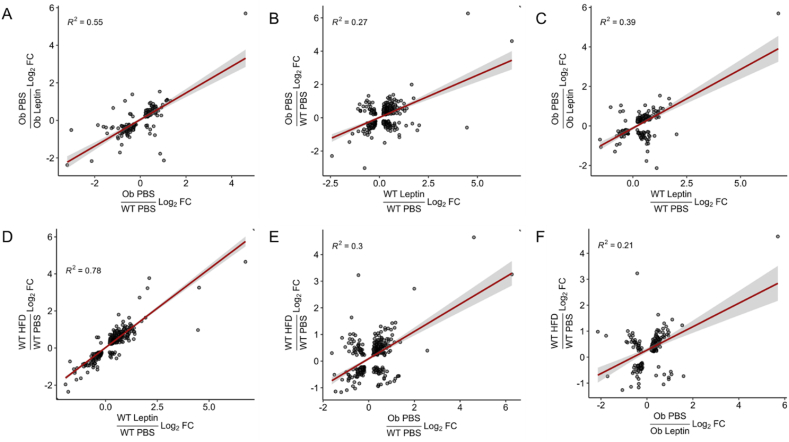
RNA-seq analysis of supernatant material from LepRb TRAP samples. Plotted are the differences in gene expression between (y-axis) (A,C) *ob/ob* mice treated with PBS vs leptin, (B) PBS-treated *ob/ob* and WT mice, or (D–F) PBS treated HFD or Chow-fed WT mice compared to (x-axis) differences in gene expression between (A, E) PBS-treated *ob/ob* and WT mice, (B–D) leptin and PBS-treated WT mice, (E) PBS-treated *ob/ob* and WT mice, (F) *ob/ob* mice treated with PBS vs leptin. For all panels, log_2_Fold Change (FC) is shown for each gene that is significantly different for both comparisons.

To determine whether DIO- and leptin-stimulated gene expression changes in the TRAP supernatant (much of which presumably derives from non-LepRb neurons) followed the same patterns as for LepRb neurons, we also compared changes in gene expression in these samples (Supplemental Tables 5–8) (Figure 2). The overlap of significantly regulated genes between groups is shown in Supplemental Fig. 1. We generally observed similar correlations in gene expression across conditions for the TRAP supernatant as in the TRAP material-leptin treatment in *ob/ob* mice altered gene expression toward that of lean wild-type mice while gene expression in DIO mice was similar to that of leptin-treated wild type mice. These findings are consistent with the idea that most leptin-stimulated changes in gene expression in the non-LepRb neurons likely represented in the TRAP supernatant sample result from leptin-stimulated changes in LepRb neurons.

Thus, gene expression in both LepRb and presumptive non-LepRb neurons from DIO animals was similar to that observed in lean animals with hyperleptinemia, suggesting that increased leptin represents the main driver of altered hypothalamic gene expression in DIO. The modest correlation between gene expression changes provoked by increased leptin under these conditions and the program promoted by physiologic leptin concentrations or leptin normalization in leptin-deficient animals suggests significant differences in the gene expression programs provoked by leptin replacement versus hyperleptinemia, however.

### 3.2 Increased leptin recapitulates DIO-associated alterations in VMN LepRb neuron electrical properties

To understand whether DIO and elevated leptin might also provoke similar changes in the activity of LepRb neurons, we chose to examine VMN LepRb neurons, which represent a more uniform population of cells (at least transcriptionally) than LepRb neurons in the ARC or DMH [7]. Furthermore, DIO-induced changes in the function of ARC and DMH neurons have been examined previously, while the effect of DIO and chronic leptin exposure on the activity of VMN LepRb neurons has remained undefined. VMN LepRb neurons are known to promote energy expenditure in response to leptin and DIO, however [10,11,41].

**Figure 3:**
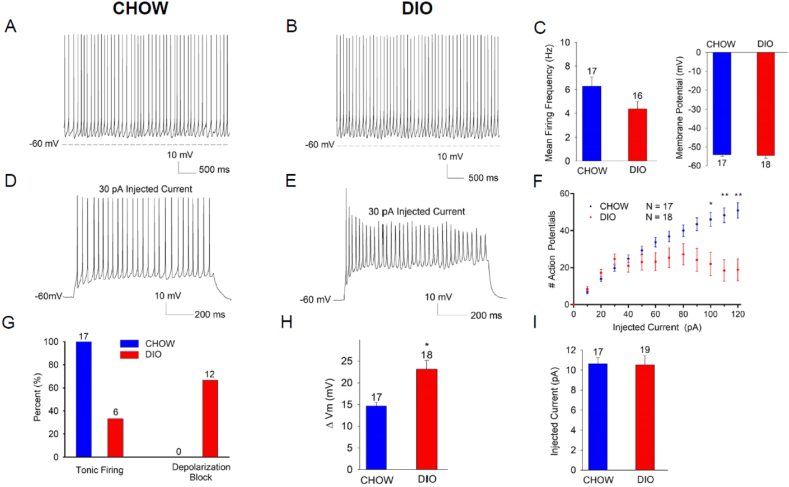
DIO alters the excitability of VMN LepRb neurons. Patch clamp electrophysiological analysis of VMN LepRb neurons from LepRb^eGFP−L10a^ mice raised on normal chow (CHOW) or HFD (DIO). (A, B) Representative current clamp recordings of membrane potential and spontaneous action potential firing for VMN LepRb neurons from CHOW (A) and DIO (B) mice. (C) Mean spontaneous firing frequency (left) and membrane potential (right) for VMN LepRb neurons from CHOW (blue; n = 17) and DIO mice (red; n = 16), p > 0.05. Neurons exhibiting a hyperpolarized membrane potential without action potential firing were excluded from analysis (0/17 cells chow fed, 2/18 cells DIO). (D,E) Representative recordings of membrane potential in response to 30 pA current injection for VMN LepRb neurons from CHOW (D) and DIO (E) mice. (F) Action potentials generated during a 1 s step of current injection from 10 to 120 pA for VMN LepRb neurons from CHOW (blue; n = 17) and DIO (red; n = 19) mice. (G) Number of VMN LepRb neurons from CHOW (blue) and DIO (red) mice that displayed tonic firing (left bars) or depolarization block (right bars), with the number neurons noted above the bar. (H) Change in Vm during 30 pA current injection for VMN LepRb neurons from CHOW (blue) and DIO (red) mice. (I) Mean rheobase current for VMN LepRb neurons from CHOW (blue) and DIO (red) mice. Data are plotted as mean +/−SEM; ∗p < 0.05, ∗∗p < 0.01 by unpaired t-test (C, H) or one-way ANOVA (F).

We used patch-clamp electrophysiology to examine electrical parameters in genetically identified VMN LepRb neurons from lean and DIO LepRb^eGFP−L10a^ mice (Figure 3). VMN LepRb neurons from lean and DIO mice displayed similar baseline membrane potential and spontaneous firing frequencies (Figure 3A–C). Furthermore, we noted no differences in cell capacitance, input resistance, or action potential parameters between groups (Supplemental Fig. 2). To assess intrinsic membrane properties, we examined voltage responses to current injections in VMN neurons from chow and DIO mice (Figure 3D–I). Neurons from DIO mice exhibited larger membrane depolarizations in response to equivalent current injections compared to controls. Despite larger changes in membrane potential, action potential firing rates were not increased at depolarizing current injections less than 60 pA. At higher current injections, however, DIO neurons displayed a reduced capacity to sustain repetitive firing and vulnerability to depolarization block. As shown in Figure 3G, all neurons from chow fed mice exhibited tonic firing and normal response to current injections (n = 17), whereas increasing current injections produced depolarization block in 67% of the neurons from DIO mice (n = 18). Rheobase (the minimal current necessary to elicit first action potential), was not different for VMN LepRb neurons from chow vs DIO mice (Figure 3I), indicating the initial activation threshold for action potential firing was not altered. Overall, these findings suggest that VMN LepRb neurons exhibit an increased voltage response to current injection in DIO mice, in some cases to the extent that the neurons undergo depolarization block. These changes are likely not due to alterations in the afterhyperpolarization (AHP) following action potential firing as we observed no difference in the fast component of the AHP and only a slight difference in the slow component of the AHPAHP for chow and DIO animals (Supplemental Fig. 3).

**Figure 4:**
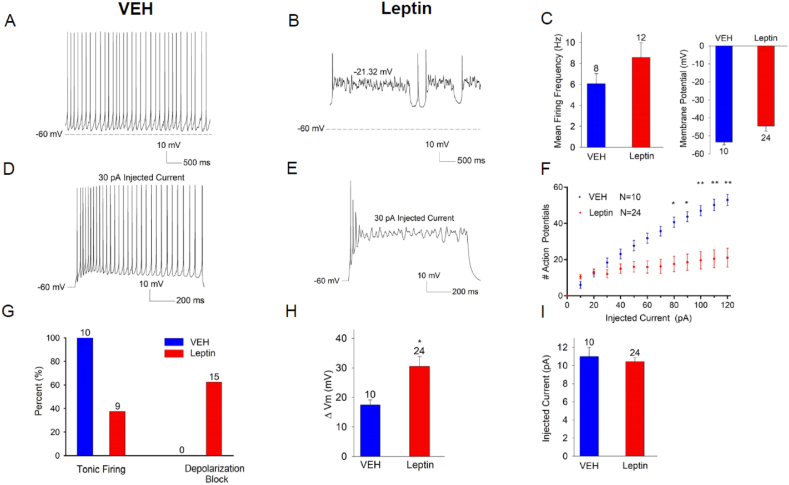
Exogenous leptin provided by minipump *in vivo* alters the excitability of VMN LepRb neurons. 10–12-week-old LepRb^eGFP−L10a^ mice raised on normal chow were implanted with minipumps containing saline vehicle (VEH) or Leptin (2.2 mg/kg per day). After 10 days, we used patch-clamp electrophysiology to examine the function of their VMN LepRb neurons. Representative current clamp recordings of membrane potential and spontaneous action potential firing for VMN LepRb neurons from VEH (A) and Leptin (B) mice. (C) Mean spontaneous firing frequency (left) and membrane potential (right) for VMN LepRb neurons from VEH (blue; n = 8 (2 cells that were hyperpolarized at baseline were excluded from the analysis)) and Leptin minipump mice (red; n = 12; cells demonstrating spontaneous firing only), p > 0.05. (D,E) Representative recordings of membrane potential in response to 30 pA current injection for VMN LepRb neurons from VEH (D) and Leptin (E) mice. (F) Action potentials generated during a 1 s step of current injection from 10 to 120 pA for VMN LepRb neurons from VEH (blue; n = 10) and Leptin (red; n = 24) mice. (G) Number of VMN LepRb neurons from VEH (blue) and Leptin (red) mice that displayed tonic firing (left bars) or depolarization block (right bars), with the number neurons noted above the bar. (H) Change in Vm during 30 pA current injection for VMN LepRb neurons from VEH (blue) and Leptin (red) mice. (I) Mean rheobase current for VMN LepRb neurons from VEH (blue) and Leptin (red) mice. Data are plotted as mean +/−SEM; ∗p < 0.05, ∗∗p < 0.01 by unpaired t-test (C, H) or one-way ANOVA (F).

To understand whether the altered electrical properties of VMN LepRb neurons in DIO mice might result from hyperleptinemia (as for DIO-mediated transcriptional changes in hypothalamic LepRb neurons), we implanted minipumps in lean chow-fed LepRb^eGFP−L10a^ mice to infuse vehicle (saline) or leptin (2.2 mg/kg per day; Leptin-MP animals) for 10 days before examining their electrical activity at baseline and in response to the injection of depolarizing current (Figure 4). Saline-treated animals gained 0.7 +/− 1.2 g of body weight during treatment, while Leptin-MP animals lost 2.8 +/− 1.2 g, consistent with elevated circulating leptin concentrations in these animals.

Under unstimulated conditions, approximately half of the cells (13/24 cells) from Leptin-MP animals displayed disrupted spontaneous firing characteristics, exhibiting burst-like firing patterns with depolarized membrane potentials (Figure 4A–B). While the remaining Leptin-MP neurons (12 non-bursting/non-depolarized cells) tended toward increased spontaneous firing rate compared to VMN LepRb neurons from control animals (8.6 Hz vs 6 Hz; Figure 4C), this effect was not statistically significant.

As for VMN LepRb neurons from DIO mice (Figure 3), input–output analysis showed that neurons from Leptin-MP animals exhibited an enhanced voltage response and an inability to sustain tonic repetitive firing with increasing levels of current injection due to depolarization block (Figure 4D–I). As shown in Figure 4G, all neurons from vehicle-treated mice exhibited tonic firing and normal response to current injections (n = 10), whereas 63% of the neurons from Leptin-MP mice exhibited depolarization block in response to increasing current injections (n = 24). As for chow and DIO mice, rheobase was not different for VMN LepRb neurons from vehicle-treated vs Leptin-MP mice. Hence, hyperleptinemia in lean mice produced alterations in the intrinsic properties of VMN LepRb neurons to a similar or greater extent than observed with endogenous hyperleptinemia in DIO mice, consistent with the notion that chronically increased leptin action on VMN LepRb neurons promotes the increased excitability of these cells.

**Figure 5:**
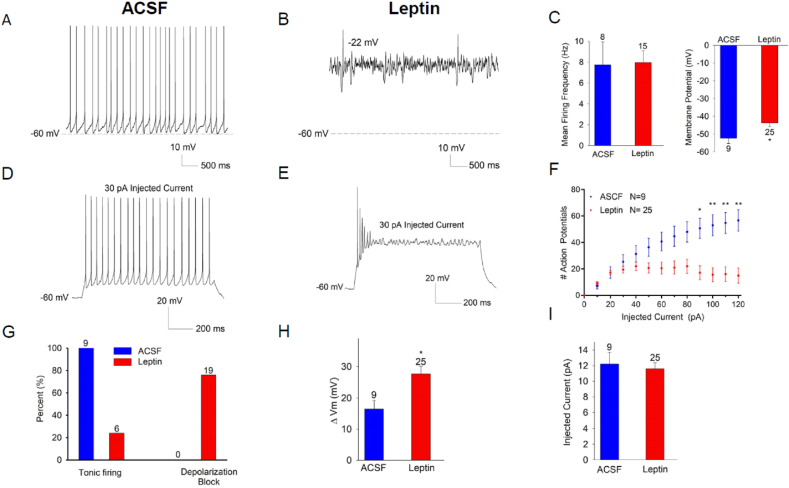
Preincubation with leptin *ex vivo* alters the excitability of VMN LepRb neurons. We prepared fresh brain slices from 10 to 12-week-old LepRb^eGFP−L10a^ lean mice, which were incubated in ACSF or ACSF containing 100 nM Leptin (Leptin) for 2–4 h prior to patch-clamp electrophysiology to examine the function of VMN LepRb neurons. Representative current clamp recordings of membrane potential and spontaneous action potential firing for VMN LepRb neurons from ACSF (A) and Leptin (B) preincubated slices. (C) Mean spontaneous firing frequency (left) and membrane potential (right) for VMN LepRb neurons from ACSF (blue; n = 8 (one cell that was hyperpolarized at baseline was excluded from analysis)) and Leptin (red; n = 15; cells demonstrating spontaneous firing only) preincubated slices, p > 0.05. Neurons exhibiting hyperpolarized membrane potential without action potential firing were excluded from analysis (1/9 cells control, 1/15 cells leptin). (D,E) Representative recordings of membrane potential in response to 30 pA current injection for VMN LepRb neurons from ACSF (D) and Leptin (E) preincubated slices. (F) Action potentials generated during a 1 s step of current injection from 10 to 120 pA for VMN LepRb neurons from ACSF (blue; n = 10) and Leptin (red; n = 25) preincubated slices. (G) Number of VMN LepRb neurons from ACSF (blue) and Leptin (red) preincubates slices that displayed tonic firing (left bars) or depolarization block (right bars), with the number neurons noted above the bar. (H) Change in Vm during 30 pA current injection VMN LepRb neurons from ACSF (blue) and Leptin (red) preincubated slices. (I) Mean rheobase current for VMN LepRb neurons from ACSF (blue) and Leptin (red) preincubated slices. Data are plotted as mean +/−SEM; ∗p < 0.05, ∗∗p < 0.01 by unpaired t-test (C, H) or one-way ANOVA (F).

To determine whether exogenous leptin can recapitulate these changes in VMN LepRb neurons *ex vivo*, we examined the potential ability of exogenous leptin to alter the electrical properties of these cells when added directly to hypothalamic slice preparations (Figure 5). This analysis revealed no changes in response to leptin application to the bath during recording over 30 min (data not shown), however. Thus, given the longer time course of tyrosine kinase- and STAT-mediated events on cell physiology and the chronic nature of leptin action, we examined the effects of preincubating the hypothalamic slices with leptin (100 nM) for longer periods of time (2–4 h) prior to recording. As for VMN LepRb neurons from Leptin-MP animals (Figure 4) we observed altered spontaneous firing characteristics (burst-like firing patterns with depolarized membrane potentials) in a subset (10 out of 25 cells) of neurons recorded from the leptin preincubated slices (Figure 5A–C). The remaining 15 VMN LepRb neurons from slices preincubated with leptin demonstrated no change in spontaneous firing rate, however (Figure 5C). There was however, a significant increase in the basal membrane potential in the VMN LepRb neurons preincubated with leptin (N = 24).

As for VMN LepRb neurons from DIO mice and Leptin-MP mice, input–output analysis showed that neurons from leptin treated slices exhibited an inability to sustain tonic repetitive firing with increasing levels of current injection due to depolarization block (Figure 5D–I). Overall, 76% of leptin treated cells exhibited depolarization block following current injection (Figure 5G; n = 19). Rheobase was not different for VMN LepRb neurons preincubated with leptin vs ACSF.

Thus, exposure to high leptin concentrations for 2–4 h *ex vivo*, like increases in leptin concentration in the absence of obesity *in vivo*, recapitulate DIO-mediated changes in the electrical properties of VMN LepRb cells, consistent with the notion that hyperleptinemia drives the observed changes in electrical activity, as well as gene expression, in hypothalamic LepRb neurons during DIO.

### 3.3 Artificial activation promotes increased VMN LepRb output in DIO mice

**Figure 6:**
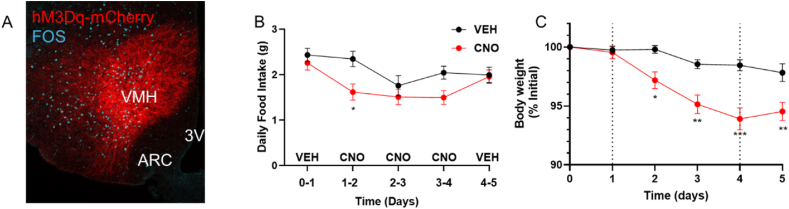
hM3Dq-mediated DREADD activation of VMN LepRb neurons promotes weight loss in DIO mice. (A) Representative image of mCherry (dsRed-immunoreactivity (-IR); red) and FOS-IR (blue) in the VMN of DIO VMN^Lepr−Dq^ animal treated with CNO (1 mg/ml, IP) 90 min prior to perfusion. (B, C). Daily food intake (B) and body weight (C) for VMN^Lepr−Dq^ animals during 3 days (days 1–4 in the graph, demarcated by dotted lines) of treatment with vehicle (VEH; 0.9% saline) or CNO (1 mg/kg, BID) (n = 8). Body weight is plotted as percentage of animals' initial weight. Data are plotted as mean +/−SEM; ∗p < 0.05, ∗∗p < 0.01, ∗∗∗p < 0.001 by unpaired t-test.

Because it is not possible to know what level of current injection in the *ex vivo* current clamp analysis might correspond to the conditions experienced by neurons *in vivo*, the foregoing analysis cannot determine whether the altered function of VMN LepRb neurons from DIO mice leads to an enhanced response to depolarizing inputs, or rather leads to depolarization block that would impair their function *in vivo*. We thus injected tTARGIT vectors into the VMN of DIO *Lepr*^*Cre*^*;Slc16a2*^*FlpO*^ mice [11] to promote the expression of the activating (hM3Dq) designer receptor exclusively activated by designer drugs (DREADD) specifically in VMN LepRb neurons (Figure 6). Treatment with CNO in these VMN^Lepr−Dq^ animals is expected to promote Gq-dependent signaling and increase intracellular calcium in VMN LepRb neurons. Consistently, CNO treatment promoted FOS accumulation in the VMH of hM3Dq-expressing mice (Figure 6A). This treatment would thus be expected to further increase the activity and output of the VMN LepRb neurons, although this should not be the case if neuronal activation and output were occluded by depolarization block *in vivo*.

We found that CNO treatment in DIO VMN^Lepr−Dq^ animals decreased food intake and body weight over a 3-day period (Figure 6B and C), suggesting that it is possible to increase the activity and output of VMN LepRb neurons in DIO mice-arguing against the notion that hyperleptinemia promotes depolarization block to impair the function in these neurons *in vivo*.

## 4 Discussion

Overall, our data reveal increased leptin signaling to the transcriptome in hypothalamic LepRb neurons during DIO; increased leptin action also recapitulates DIO-like changes in the electrical activity of VMN LepRb neurons. Together, these data reveal increased cellular leptin action in LepRb neurons during DIO. Thus, obesity in DIO mice does not result from impaired LepRb signaling; rather, the hyperleptinemia of DIO drives increased LepRb signaling and cellular leptin action.

The finding that cellular leptin action is increased, rather than decreased, in hypothalamic LepRb neurons of DIO mice is consistent with the observation that the baseline pSTAT3 signal (representing the response to endogenous circulating leptin) is increased in the ARC of DIO mice relative to lean controls [19,31,42]. Similarly, others have demonstrated the ongoing suppression of food intake by endogenous leptin in DIO mice [43]. Thus, endogenous LepRb signaling in hypothalamic LepRb neurons is increased (not decreased) in DIO, demonstrating that obesity does not result from “leptin resistance” (where this term is given to mean decreased LepRb signaling due to HFD-feeding and/or obesity) [44]. In addition to countering the hypothesis that attenuated LepRb signaling in DIO causes obesity, these findings argue against the idea that obesity results from lower CNS leptin concentrations (e.g., due to impaired leptin transport into the CNS).

Although our data reveal hyperleptinemia can mediate effects on LepRb neuron gene expression in DIO mice, the pSTAT3 response to exogenous leptin is blunted in DIO relative to lean mice [19,21,45,46]. Thus, while LepRb signaling in response to endogenous leptin is increased in DIO compared to lean animals, consistent with their increased circulating leptin concentrations, processes that restrain LepRb signaling must operate in DIO, limiting the maximal signaling response to acute supraphysiologic leptin administration [47].

Importantly, hyperleptinemia itself plays a role in limiting leptin action in DIO. Restoring normal leptin concentrations by minipump implantation in leptin-deficient *ob/ob* mice renders these “leptin-clamped” mice lean, but these mice can be rendered obese by high-calorie diet without altering their leptin concentrations [33]. Unlike hyperleptinemic DIO mice, the leptin-clamped DIO *ob/ob* mice remain leptin sensitive (despite their obesity), although artificial hyperleptinemia can render mice insensitive to the anorectic effects of exogenous leptin. Thus, increased leptin itself limits additional leptin action.

Leptin may limit its own action in several ways, including by increasing the expression of cellular inhibitors of LepRb signaling [48,49]. Indeed, our present analysis revealed that the expression of *Socs3* in hypothalamic LepRb neurons increased by > 3-fold and *Ptpn1* (which encodes PTP1B) and *Ptpn2* (which encodes TCPTP) increased 1.2-fold in LepRb neurons in DIO relative to lean control mice, consistent with the known regulation of these genes by leptin *in vivo* [22,23,28]. While the induction of signaling inhibitors by leptin is unlikely to decrease LepRb signaling below baseline in DIO animals, it would be expected to limit the incremental augmentation of LepRb signaling in response to increased leptin concentrations.

DIO and exogenous leptin induced similar alterations in the electrical properties of VMN LepRb neurons, rendering them more sensitive to activating inputs. In addition, leptin treated animals and slices displayed basal membrane depolarization in a subset of VMN LepRb neurons. While this difference may reflect mechanistically separable pathways by which exogenous leptin and DIO alter VMN LepRb function, the ability of exogenous leptin to exert similar (albeit larger) changes than endogenous hyperleptinemia in DIO represents most parsimonious explanation for these findings. Because exogenous leptin cannot precisely duplicate the complex brain response to DIO, it is possible that other adaptations (e.g. changes in synaptic input), may restrain the direct effect of leptin on VMN LepRb neurons. Alternatively, chronic endogenous hyperleptinemia during DIO may engage homeostatic mechanisms that normalize baseline membrane potential and firing rates over time, masking tonic depolarizing effects.

Previous studies demonstrated a heterogeneous response of VMN neurons to acute leptin exposure, including a subset of cells exhibiting leptin-induced hyperpolarization [10,50,51]. While these observations raise the possibility of decreased VMN LepRb activity in the face of chronic leptin, we found no difference in the number of cells exhibiting hyperpolarization without action potential firing in control vs DIO or leptin treatment under basal conditions, and our predominant finding was augmented increases in Vm in response to depolarizing input.

While our *ex vivo* electrophysiology data cannot determine whether the DIO- and leptin-induced changes in the intrinsic properties of VMN LepRb neurons increase the *in vivo* activity of these cells during DIO (or rather inhibit their activity due to depolarization block), activating VMN LepRb cells decreases food intake increases energy expenditure and LepRb expression on these neurons is required to mediate responses to DIO [10,11]. Thus, increased leptin action on these cells during DIO presumably increases their activity *in vivo*. Indeed, chemogenetically activating VMN LepRb neurons in DIO animals decreases food intake and body weight, suggesting that these cells are not rendered non-functional due to depolarization block during DIO *in vivo*. Because we did not measure other parameters, it remains possible that some classes of VMN LepRb neurons that potentially modulate other aspects of physiology (such as energy expenditure and related parameters), might not be altered by the DREADD-mediated activation of VMN LepRb neurons, however. While increased cellular leptin action appears to promote beneficial effects via VMN LepRb neurons, it is possible that excess leptin action on other cell types mediates negative metabolic outcomes, however.

Our TRAP-seq data reveal that a significant component of the transcriptional response to physiological leptin (*i.e.*, in lean compared to leptin-deficient mice [37]) differs from the response to elevated leptin (as with exogenous leptin administration or in DIO), suggesting that important aspects of the response to excess leptin differ in both effect and magnitude from the response to leptin restoration in leptin-deficient states. Furthermore, our finding of increased cellular leptin action is consistent with the notion that the increased LepRb signaling in DIO animals may not be metabolically beneficial or even benign. Indeed, rodent models of excess (overexpressed) leptin display obesity under some conditions [52], and excess leptin may contribute to the genesis of hypertension and impaired glycemic control [18,31,32]. Furthermore, several recent studies suggest that, although physiologic leptin levels prevent the hyperphagia and obesity associated with leptin deficiency [43], the increased leptin concentrations in obese animals may actually promote and/or augment obesity and diabetes [34,35].

Given that leptin signals via a receptor tyrosine kinase (rather than via a GPCR), it is not surprising that leptin-induced changes in the electrical activity of VMN LepRb neurons require substantial exposure time. This requirement for longer exposure times is consistent with the chronic nature of leptin action; this finding also suggests that cellular processes with long time courses contribute to leptin-stimulated changes in electrical parameters. These could include changes in gene expression and/or other tyrosine kinase-mediated changes in cell physiology. If leptin were to promote different electrical activity-controlling gene expression programs in distinct populations of LepRb neurons, this could explain leptin's ability to decrease the activity of some LepRb cells, while activating others. In the future, it will be interesting to examine the control of gene expression in individual populations of hypothalamic LepRb neurons rather than for all LepRb cell types together, in order to identify potential mediators of leptin- and DIO-induced changes in each LepRb cell type.

Overall, our present findings demonstrate that DIO represents a state of elevated leptin→LepRb signaling in hypothalamic LepRb neurons. Hence, it will be important to examine the roles and mechanisms of action for leptin in the myriad changes in hypothalamic physiology that accompany DIO, as well as to understand any potential physiologic and pathophysiologic effects of excess LepRb signaling in obese animals.

## CRediT authorship contribution statement

**James Dell'Orco:** Writing – review & editing, Methodology, Investigation, Formal analysis, Conceptualization. **Warren Pan:** Writing – review & editing, Investigation, Formal analysis, Conceptualization. **Margaret B. Allison:** Writing – review & editing, Investigation, Formal analysis, Conceptualization. **Abigail J. Tomlinson:** Writing – review & editing, Investigation, Formal analysis. **Jordan Wean:** Writing – review & editing, Formal analysis. **Paul V. Sabatini:** Writing – review & editing, Investigation, Formal analysis, Conceptualization. **Christopher J. Rhodes:** Writing – review & editing, Resources, Conceptualization. **David P. Olson:** Writing – review & editing, Resources, Conceptualization. **Martin G. Myers:** Writing – review & editing, Writing – original draft, Supervision, Resources, Funding acquisition, Conceptualization. **Paulette B. Goforth:** Writing – review & editing, Supervision, Methodology, Investigation, Formal analysis, Conceptualization.

## Declaration of competing interest

CJR is a former employee of and owns stock in AstraZeneca; MGM receives research funding from AstraZeneca, Eli Lilly, and Novo Nordisk, and consults for and/or has received honoraria from Tenvie, Pfizer, Novo Nordisk, and Zealand Pharmaceuticals. DPO and PBG receive research funding from Eli Lilly. The authors declare that they have no other conflicts of interest relevant to this manuscript.

## Data Availability

Data will be made available on request.
