# Supplementary material for: Increased leptin signaling drives the response of hypothalamic LepRb neurons to diet-induced obesity
Source: Mol Metab. 2026 May 2;108:102378. doi: 10.1016/j.molmet.2026.102378 (PMC13206730; doi:10.1016/j.molmet.2026.102378)
Supplement: Multimedia component 9 [file mmc9.pdf]

Supplemental Data

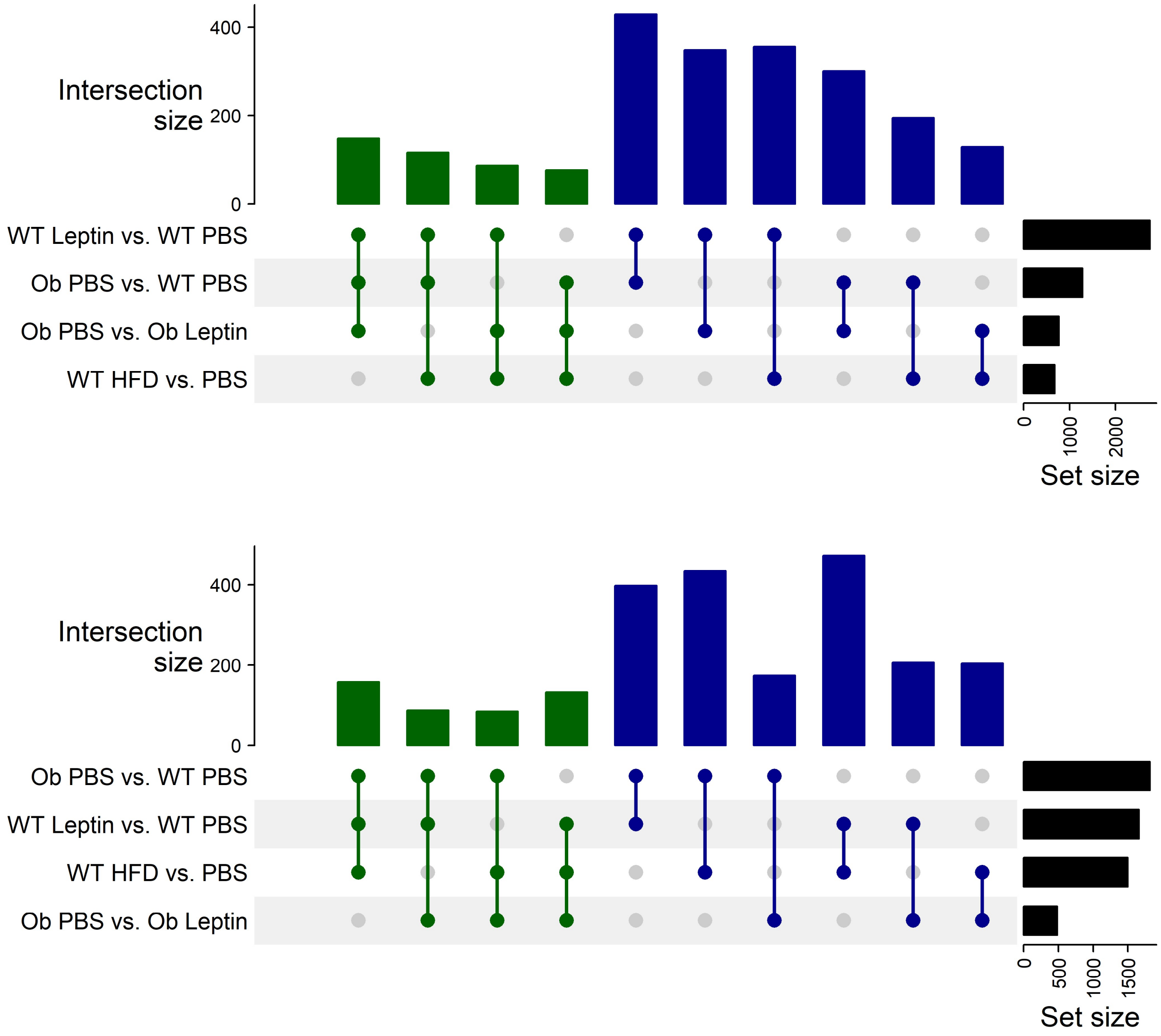

Supplemental Figure 1. Overlap of significantly regulated genes between conditions (Intersection size) and number of significantly regulated genes (Set size) for each comparison for LepRb TRAP-seq data (top panel) and RNA-seq of supernatant (bottom panel).

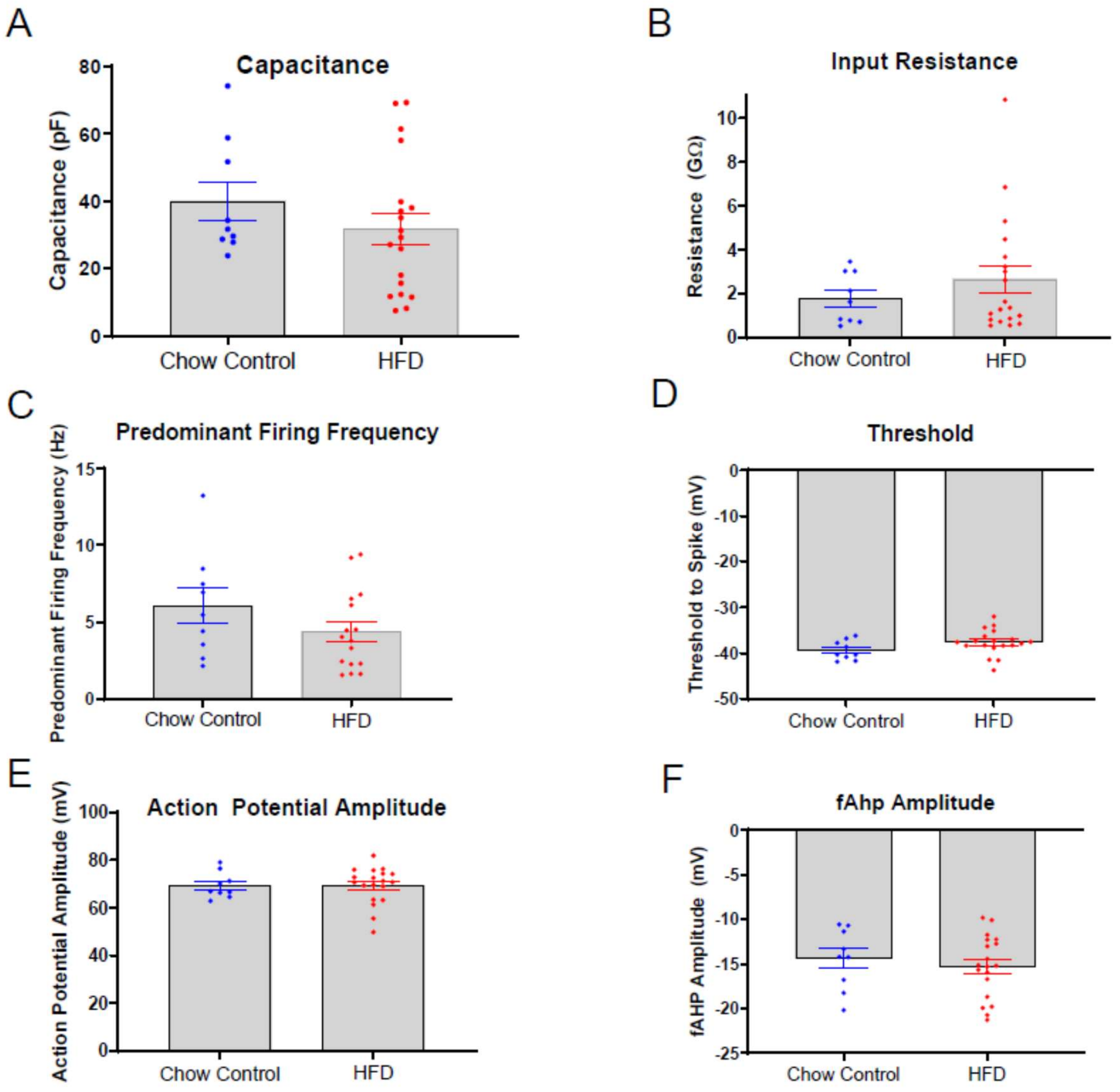

Supplemental Figure 2. Baseline electrical characteristics for VMN LepRb neurons from Chow Control and HFD-fed mice. Shown are (A) mean capacitance, (B) mean input resistance, (C) mean action potential threshold, (D) mean threshold to spike, (E) mean peak action potential voltage, and (F) mean peak amplitude of the fast component of the afterhyperpolarization (AHP) phase of the action potentials. Data are plotted as mean -/+ SEM; all comparisons not significantly different.

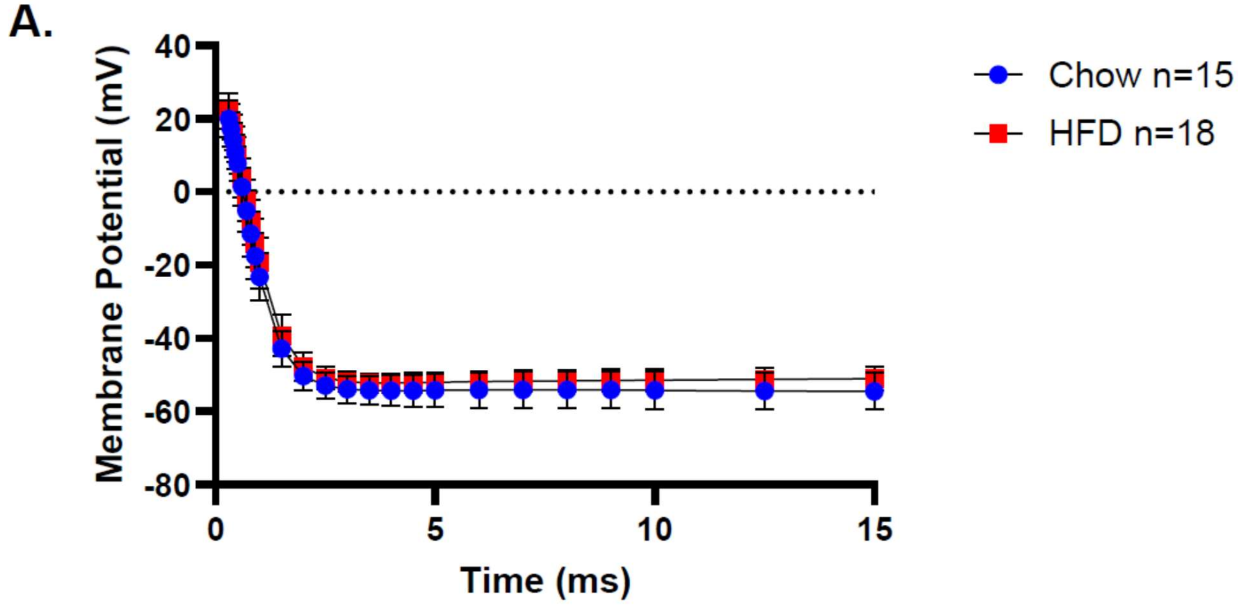

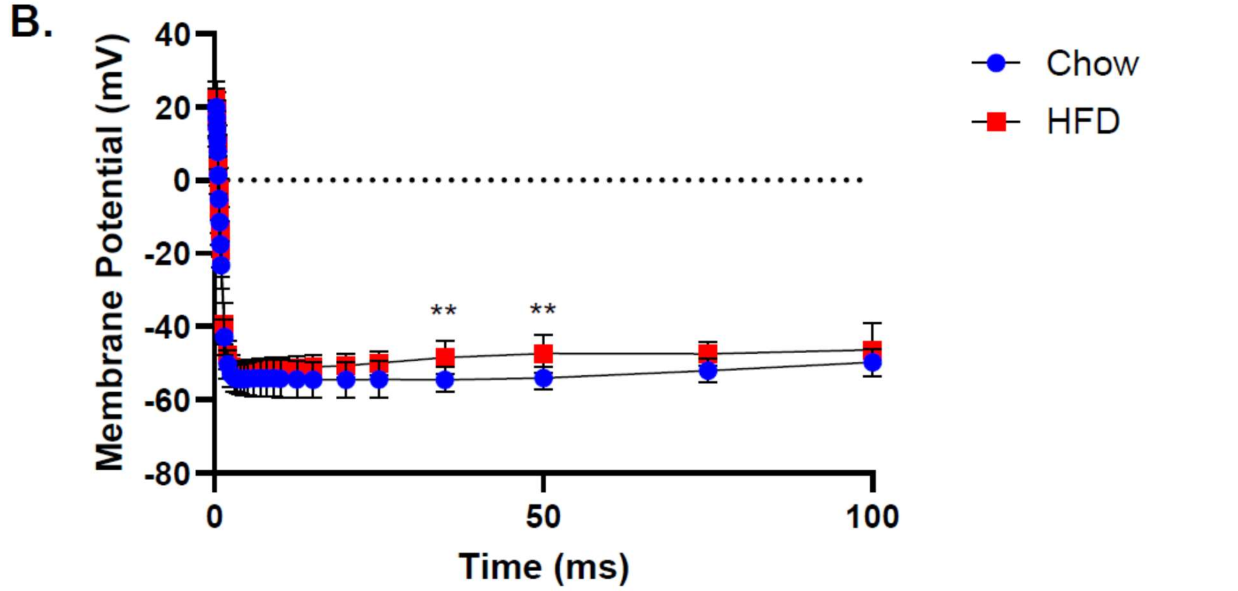

Supplemental Figure 3. Afterhyperpolarization analysis of hypothalamic LepRb neurons in the context of chow and high fat diet. Comparison of the afterhypolarization (AHP) phase of the action potential showing (A) the average fast component of the AHP measured within 15 milliseconds of the peak, and (B) average slow component of the

AHP measured out to 100 milliseconds after the peak. Data are plotted as mean -/+ SD; ** by Two-way ANOVA.
